# Integration of a single-step genome-wide association study with a multi-tissue transcriptome analysis provides novel insights into the genetic basis of wool and weight traits in sheep

**DOI:** 10.1186/s12711-021-00649-8

**Published:** 2021-06-30

**Authors:** Bingru Zhao, Hanpeng Luo, Xixia Huang, Chen Wei, Jiang Di, Yuezhen Tian, Xuefeng Fu, Bingjie Li, George E. Liu, Lingzhao Fang, Shengli Zhang, Kechuan Tian

**Affiliations:** 1 National Engineering Laboratory for Animal Breeding, Key Laboratory of Animal Genetics, Breeding, and Reproduction, Ministry of Agriculture, College of Animal Science and Technology, China Agricultural University, Beijing, China; 2 College of Animal Science, Xinjiang Agricultural University, Urumqi, China; 3 Key Laboratory of Genetics Breeding and Reproduction of the Fine Wool Sheep & Cashmere Goat in Xinjiang, Institute of Animal Science, Xinjiang Academy of Animal Sciences, Urumqi, China; 4 Scotland’s Rural College (SRUC), Roslin Institute Building, Midlothian, EH25 9RG UK; 5 Animal Genomics and Improvement Laboratory, Henry A. Wallace Beltsville Agricultural Research Center, Agricultural Research Service, Agricultural Research Service, USDA, Beltsville, MD USA; 6 MRC Human Genetics Unit at the Institute of Genetics and Cancer, University of Edinburgh, Edinburgh, UK; 7 Institute of Animal Science and Veterinary Medicine, Shandong Academy of Agricultural Sciences, Jinan, China

## Abstract

**Background:**

Genetic improvement of wool and growth traits is a major goal in the sheep industry, but their underlying genetic architecture remains elusive. To improve our understanding of these mechanisms, we conducted a weighted single-step genome-wide association study (WssGWAS) and then integrated the results with large-scale transcriptome data for five wool traits and one growth trait in Merino sheep: mean fibre diameter (MFD), coefficient of variation of the fibre diameter (CVFD), crimp number (CN), mean staple length (MSL), greasy fleece weight (GFW), and live weight (LW).

**Results:**

Our dataset comprised 7135 individuals with phenotype data, among which 1217 had high-density (HD) genotype data (n = 372,534). The genotypes of 707 of these animals were imputed from the Illumina Ovine single nucleotide polymorphism (SNP) 54 BeadChip to the HD Array. The heritability of these traits ranged from 0.05 (CVFD) to 0.36 (MFD), and between-trait genetic correlations ranged from − 0.44 (CN vs*.* LW) to 0.77 (GFW vs. LW). By integrating the GWAS signals with RNA-seq data from 500 samples (representing 87 tissue types from 16 animals), we detected tissues that were relevant to each of the six traits, e.g. liver, muscle and the gastrointestinal (GI) tract were the most relevant tissues for LW, and leukocytes and macrophages were the most relevant cells for CN. For the six traits, 54 quantitative trait loci (QTL) were identified covering 81 candidate genes on 21 ovine autosomes. Multiple candidate genes showed strong tissue-specific expression, e.g. *BNC1* (associated with MFD) and *CHRNB1* (LW) were specifically expressed in skin and muscle, respectively. By conducting phenome-wide association studies (PheWAS) in humans, we found that orthologues of several of these candidate genes were significantly (FDR < 0.05) associated with similar traits in humans, e.g. *BNC1* was significantly associated with MFD in sheep and with hair colour in humans, and *CHRNB1* was significantly associated with LW in sheep and with body mass index in humans.

**Conclusions:**

Our findings provide novel insights into the biological and genetic mechanisms underlying wool and growth traits, and thus will contribute to the genetic improvement and gene mapping of complex traits in sheep.

**Supplementary Information:**

The online version contains supplementary material available at 10.1186/s12711-021-00649-8.

## Background

Chinese Merino is a dual-purpose sheep breed that is widespread in Northwest China and renowned for its environmental adaptability and high-quality wool and mutton [1]. The genetic improvement of complex traits that are relevant to wool and mutton production is essential in the sheep industry [2]. Although the genetic variation of such economic traits has been explored [3–5], the genetic architecture underlying the control of wool and growth traits is not fully elucidated, which hinders genetic improvement programmes.

Genome-wide association studies (GWAS) have become an efficient approach to identify single nucleotide polymorphisms (SNPs) that are associated with complex traits in humans and livestock [6–8]. Several single-marker GWAS of wool and growth traits in sheep have been conducted [9–11]. For instance, Wang et al. [9] reported 12 candidate genes for wool traits in Merino sheep (n = 765). Ebrahimi et al. [10] found three significant SNPs associated with greasy fleece weight in a population of 96 Baluchi sheep, and Bolormaa et al. [11] studied 22 traits, including wool and breech conformation traits, in a population of 5726 Merino and crossbred sheep. In addition to GWAS, the analysis of selection signatures is also commonly used to detect genetic associations with wool traits in sheep [3, 5]. For instance, Megdiche et al. [5] found that genomic regions under positive selection in Merino and other Merino-derived breeds were significantly associated with wool traits. However, these analyses were limited by the number of animals for which both genotypes and phenotypes were available. The weighted single-step genome-wide association study (WssGWAS) approach was derived from the single-step genomic best linear unbiased prediction (ssGBLUP) method [12, 13]. Compared with the classical single-marker GWAS, WssGWAS allows the simultaneous use of all data, including those from individuals with phenotype but without genotype data, by using a scaled and properly augmented relationship matrix ($\mathbf{H}$ matrix). This efficient approach for identifying genes or quantitative trait loci (QTL) that underlie complex traits in animals has recently emerged [14–16]. In addition, WssGWAS enables SNPs to be weighted differently and multiple markers to be tested jointly via a sliding window strategy [17]. Based on these properties, WssGWAS might be able to provide more accurate estimates of genetic parameters than the classical GWAS, thereby leading to an increased power of QTL detection [18–20].

Although the GWAS approach has been useful for discovering trait-associated genomic variants, the causal tissues and cell types that are affected by such variants are largely unknown [21]. The discovery of tissues and cell types that are relevant to complex traits is critical for understanding the genetic regulatory mechanisms that underlie the control of such traits [22]. The integration of a GWAS with a multi-tissue transcriptome analysis offers the potential to dissect causal tissues and cell types for complex traits [21, 23]. For instance, by integrating multiple-tissue eQTL, the GTEx Consortium [24] highlighted the tissues that are genetically responsible for complex traits in humans, such as the brain for schizophrenia and age of puberty onset. By combining the transcriptome of 91 tissues with the GWAS results of 45 complex traits in cattle, Fang et al. [21] revealed candidate tissues and genes for several traits, such as the blood/immune tissues for male fertility traits. More recently, phenome-wide association analysis (PheWAS), which is a complementary approach to GWAS, has been used to associate certain genetic variants with many phenotypes to study their pleiotropy and causality among big data [25]. Orthologous genes often show similar functions across species. Therefore, the use of rich GWAS data from humans to conduct a PheWAS might contribute to improve the characterization of the pleiotropic effects of candidate genes and elucidate the genetic architecture of complex traits in the target species [26].

The objectives of our study were: (1) to estimate the genetic parameters (e.g., heritability and genetic correlation) for five wool traits (mean fibre diameter, MFD; coefficient of variation of the fibre diameter, CVFD; crimp number, CN; mean staple length, MSL; and greasy fleece weight, GFW); and one growth trait (live weight, LW) in a dual-purpose Merino sheep population (n = 7135); (2) to identify tissues and genes that are associated with these traits by integrating the results of a WssGWAS with data from 500 RNA-seq samples of 87 tissues; and (3) to explore whether the results from human studies can help validate and explain the findings in this sheep study via a PheWAS.

## Methods

### Phenotype and pedigree data

The Merino sheep included in this study were maintained at the Xinjiang Fine Wool Sheep Breeding Farm (Xinjiang, China). Farm management of all the animals was previously described in [27]. In total, 7135 animals from 50 flocks with phenotypic records on six traits, including four wool quality traits, one wool production trait and one growth trait, were available. All phenotypes were measured once on 15-month-old females from 2012 to 2019. A detailed summary of the phenotypic records for each trait is in Table 1. Records that were more than three standard deviations (SD) from the mean were removed.

**Table 1:** Estimates of variance components and heritabilities for wool traits and live weight in Merino sheep

Trait (unit)	Number	Mean	SD	${\sigma }_{a}^{2}$	${\sigma }_{e}^{2}$	${h}^{2}$ (SE)
MFD (µm)	5436	18.24	2.07	1.04	1.87	0.36 (0.04)
CVFD (%)	5414	21.98	2.80	0.32	6.44	0.05 (0.02)
CN (/2.5 cm)	5208	12.71	2.45	0.37	4.91	0.07 (0.03)
MSL (cm)	7135	10.26	0.98	0.22	0.58	0.27 (0.03)
GFW (kg)	6727	3.95	0.62	0.08	0.22	0.28 (0.03)
LW (kg)	6871	36.56	4.99	6.07	12.23	0.33 (0.03)

SD, standard deviation; ${\sigma }_{a}^{2}$, additive variance; ${\upsigma }_{e}^{2}$, residual variance; *h*^*2*^, heritability; SE, standard error. MFD, mean fibre diameter; CVFD, coefficient of variation of the fibre diameter; CN, crimp number; MSL, mean staple length; GFW, greasy fleece weight; LW, live weight

Each wool quality trait was measured according to the standardized method established by the China Fibre Inspection Bureau (CFIB) and the International Wool Textile Organization (IWTO) [28]. Briefly, a wool sample (approximately 70 to 80 g) was collected from the right mid-side of each animal prior to shearing. The samples were sent to a commercial laboratory for measurement of a range of wool traits. Approximately 20 staples from each mid-side sample were randomly sub-sampled to measure the staple length (SL) and mean fibre crimp number (CN, per 2.5 cm). The rest of each sample was washed with detergent in hot water, rinsed twice with cold water, spun and oven-dried at 105 °C. Prior to conditioning at 20 °C and a relative humidity of 65% for 24 h, 2-mm snippets were taken from each dried sample via mini-coring to measure MFD and CVFD using an OFDA2000 instrument (BSC Electronics). At shearing, GFW, including the unskirted fleece and belly wool, of each animal was weighed. Following yearling shearing, the LW of each animal was measured.

The complete pedigree (13,528 animals) was used to construct the relationship matrix, which included 413 sires, 6476 dams and 483 yearling females.

### Genotype data

Genomic DNA was extracted from the blood samples of 1217 randomly selected female phenotypic sheep using the phenol–chloroform method. Among these, 707, 257 and 253 individuals were genotyped using the Illumina Ovine SNP54 BeadChip (Illumina Inc., San Diego, CA, USA), the Illumina Ovine SNP600 BeadChip (Illumina Inc., San Diego, CA, USA), and the Sheep 600 K Genotyping Array (Affymetrix Inc., Santa Clara, CA, USA), respectively. The reference population for genotype imputation included 510 individuals genotyped with the 600 K arrays (n = 459,467 SNPs). The target population included 707 individuals genotyped with the Illumina Ovine SNP54 BeadChip (n = 34,715 SNPs). The physical positions of SNPs were based on the sheep reference genome assembly Oar_v3.1 [29]. Genotype imputation was performed using the software BEAGLE version 5.1 [30]. Quality control of the SNPs was performed with the PLINK software [31]: a SNP was removed if its call rate was lower than 90%, its minor allele frequency (MAF) lower than 1%, if it significantly deviated from the Hardy–Weinberg equilibrium (*P* < 10^−6^) [32], if its genomic position was unknown, or if it was located on a sex chromosome. Individuals with an average call rate lower than 90% were also removed. Finally, 372,534 SNPs for 1217 individuals remained for further analyses.

### Estimation of genetic parameters

A multi-trait animal model was used to estimate (co)variance components using the average information REML procedure in the DMU package [33]:

$${\mathbf{y}} = {\mathbf{X}} {\varvec{\upbeta }} + {\mathbf{Za}} + {\mathbf{e}},$$

where **y** is the vector of phenotypes for MFD, CVFD, CN, MSL, GFW and LW; **β** is the vector of fixed effects, including flocks (50 levels), years of birth (8 levels: 2011–2018) and seasons (2 levels: spring and winter); **a** is the vector of random additive genetic effects of animals, where $\mathbf{a}\sim \text{N}(0,{\mathbf{A}{\upsigma }}_{\text{a}}^{2})$, **A** representing the pedigree-based relationship matrix and ${{\upsigma }}_{\text{a}}^{2}$ the additive genetic variance; **e** is a vector of random residuals where $\mathbf{e}\sim \text{N}\left(0,\mathbf{I}{{\upsigma }}_{\text{e}}^{2}\right)$, **I** representing the identity matrix and ${{\upsigma }}_{\text{e}}^{2}$ the residual variance; and **X** and **Z** are the corresponding incidence matrices.

Heritability was defined as ${\text{h}}^{2}=\frac{{{\upsigma }}_{\text{a}}^{2}}{{{\upsigma }}_{\text{p}}^{2}}$, ${{\upsigma }}_{\text{p}}^{2}={{\upsigma }}_{\text{a}}^{2}{+{\upsigma }}_{\text{e}}^{2}$. The square of the standard error (SE) for the estimates of heritability, the genetic correlation coefficient and the square of the SE for the genetic correlation coefficient were calculated as previously described [34].

### WssGWAS

We performed an association study using the single-step genomic BLUP (ssGBLUP) approach [19]. Genomic estimated breeding values (GEBV) of all the animals were estimated and transformed into SNP effects using the BLUPF90 family software [35]. The variance components were estimated using the AIREMLF90 module. Then, the GEBV and SNP effects were obtained using the postGSf90 module.

The single-trait animal model for ssGBLUP was as follows:

$${\mathbf{y}} = {\mathbf{X}}{\varvec{\upbeta}} + {\mathbf{Za}} + {\mathbf{e}},$$

where **y** is the vector of phenotypic observations; **β** is the vector of the same fixed effects as mentioned above; **a** is the vector of additive genetic effects and assumes that $\mathbf{a}\sim \text{N}(0,{\mathbf{H}{\upsigma }}_{\text{a}}^{2})$, where **H** is the matrix of pedigree and genomic information and ${{\upsigma }}_{\text{a}}^{2}$ is the additive genetic variance; $\mathbf{e}$ is the vector of random residuals and assumes that $\mathbf{e}\sim \mathbf{N}\left(0,\mathbf{I}{{\upsigma }}_{\mathrm{e}}^{2}\right)$, where **I** is the identity matrix and ${{\upsigma }}_{\text{e}}^{2}$ is the residual variance; and **X** and **Z** are the incidence matrices of **β** and **a**, respectively.

To solve the mixed model equations, the inverse of the **H** matrix (**H**^**−1**^) was defined as follows [36]:

$${\mathbf{H}}^{{ - 1}} = {\mathbf{A}}^{{ - 1}} + \left[ {\begin{array}{*{20}c} 0 & 0 \\ 0 & {{\mathbf{G}}_{{\mathbf{w}}} ^{{ - 1}} - {\mathbf{A}}_{{22}}^{{ - 1}} } \\ \end{array} } \right],$$

where **A** is the numerator relationship matrix applied for all pedigreed animals; **A**_**22**_ is the numerator relationship matrix applied for genotyped animals; and **G**_**w**_ is the genomic relationship matrix, which assumes the allele frequency of the current population and adjusts for compatibility with **A**_**22**_ [37].

The **G** matrix was calculated as follows:

$${\mathbf{G}} = {\mathbf{ZDZ}}^{{{\mathbf{ - 1}}}} {\text{q,}}$$

where **Z** is the marker matrix ($\text{aa}=0$; $\text{Aa}=1$, and $\text{AA}=2)$; **D** is the diagonal matrix of weights for SNP variances (initially **D** = **I**), and $\text{q}$ is the weighting factor. In this study, the weighting factor was derived by ensuring that the average diagonal in **G** was close to that of **A**_**22**_ [38].

Estimates of the SNP effects and weights for the WssGWAS were obtained by performing the following steps [12]:

1. In the first iteration $(\mathrm{t}=1), \, {\bf{D}}={\bf{I}}$; ${\mathbf{G}}_{(\text{t})}={\mathbf{Z}\mathbf{D}}_{(\text{t})}\mathbf{Z}^{\mathbf{\prime}}{\uplambda }$, where ${\uplambda }$ is a normalization constant or a variance ratio, ${\uplambda }=\frac{{{\upsigma }}_{\text{u}}^{2}}{{{\upsigma }}_{\text{a}}^{2}}$ =$\frac{1}{{\sum }_{\text{i}-1}^{\text{M}}2{\text{p}}_{\text{i}}(1-{\text{p}}_{\text{i}})}$;
2. The GEBV is calculated for the entire data set using ssGBLUP;
3. The SNP effects ($\widehat{\text{u}}$) are calculated according to the GEBV: ${\widehat{\mathbf{u}}}_{(\text{t})}={\uplambda }{{\mathbf{D}}_{(\text{t})}\mathbf{Z}}^{\prime}{\mathbf{G}}_{(\text{t})}^{-1}{\hat{\mathbf{a}}}_{{\text{g}}}$, where ${\hat{\mathbf{a}}}_{{\text{g}}}$ is the GEBV of animals that were also genotyped;
4. The weight of each SNP is calculated: ${\text{d}}_{\text{i}(\text{t}+1)}={\widehat{\text{u}}}_{\text{i}(\text{t})}^{2}2{\text{p}}_{\text{i}}(1-{\text{p}}_{\text{i}})$, where $\text{i}$ is the $\text{i}$-th SNP;
5. The SNP weights are normalized to keep the total genetic variance constant: ${\mathbf{D}}_{{\left( {{\text{t}} + 1} \right)}} = \frac{{{\text{tr}}\left( {{\mathbf{D}}_{{\left( 1 \right)}} } \right)}}{{{\text{tr}}\left( {{\mathbf{D}}_{{\left( {{\text{t}} + 1} \right)}} } \right)}}{\mathbf{D}}_{{\left( {{\text{t}} + 1} \right)}}$;
6. The weighted matrix $\bf{G}$ is calculated: ${\mathbf{G}}_{(\text{t}+1)}={\mathbf{Z}\mathbf{D}}_{(\text{t}+1)}\mathbf{Z}^{\mathbf{\prime}}{\uplambda }$; and
7. Loop back to step 2.

Iterations increased the weights of SNPs with large effects while it decreased those with small effects [13]. Thus, the procedure was run for one iteration based on the accuracies of the GEBV in the study. The percentage of genetic variance explained by the $\text{i}$-th set of consecutive SNPs ($\text{i}$-th SNP window including 20 consecutive SNPs) was calculated as follows [13]:

$$\frac{{\text{var} \left( {{\text{a}}_{{\text{i}}} } \right)}}{{\upsigma _{{\text{a}}}^{2} }} \times 100\% = \frac{{{\text{var}}\left( {\sum\nolimits_{{{\text{j}} = 1}}^{{\text{n}}} {{\mathbf{z}}_{{\text{j}}} \hat{\text{u}}_{{\text{j}}} } } \right)}}{{\upsigma _{{\text{a}}}^{2} }} \times 100\% ,$$

where ${\text{a}}_{\text{i}}$ is the genetic value of the $\text{i}$-th SNP window; ${{\upsigma }}_{\text{a}}^{2}$ is the total additive genetic variance; ${\mathbf{z}}_{{\text{j}}}$ is the vector of the genotypes of the $\text{j}$-th SNP for all individuals; and ${\hat{\text{u}}_{{\text{j}}}}$ is the genetic effect of the $\text{j}$-th SNP within the $\text{i}$-th SNP window.

### Detection of top SNP windows and functional annotations of candidate genes

For the detection of candidate genes, we arbitrarily selected the top 10 ranked windows in terms of their explained genomic variance as QTL regions for each trait. For the GO functional enrichment analysis, we used genes that were within the top 1% of the ranked windows for each trait. Previously reported sheep QTL were obtained from the Sheep QTLdb [39]. The extent of LD between SNPs was estimated using PLINK [31], and haplotype blocks were identified in the adjacent windows using Haploview 4.1 and its default parameters [40]. We used the R package ‘BiomaRt’ in Ensembl [41] to obtain information regarding the gene annotations of the ovine reference genome Oar_v3.1. Functional enrichment analysis of the gene lists for each trait was conducted using the R package clusterProfiler [42].

### Integrative analysis of GWAS and the sheep expression atlas

We collected transcriptome data from 500 ovine samples reported by Clark et al. [43], which represented 87 tissues and cell types. Briefly, these 500 samples were collected from 16 individuals at three developmental stages, including three male and three female adult sheep, two male and two female lambs, and three male and three female embryos. According to the available knowledge on tissue biology [43], these 87 tissues and cell types were classified into 13 organ systems. The details of the RNA-seq sample classification are in Additional file 1: Table S1.

The expression levels were normalized by transcripts per kb of exon model per million mapped reads (TPM). To detect genes that have a high expression in specific tissues, we used the following approach for each gene in each tissue [21, 22]:

$${\mathbf{y}} = {{\upmu}} + {\mathbf{X}}{{\upbeta}} + {\mathbf{Z}}{\text{a}} + {\mathbf{e}},$$

where **y** is the scaled log_2_ TPM; ${\upmu }$ is the intercept; **X** is the dummy variable for the tissue, where the samples of the tissue tested (e.g., immune) are denoted as ‘1’ and samples outside the organ system (e.g., nonimmune tissues and cell types) are denoted as ‘−1’; ${\upbeta }$ is the corresponding tissue effect; **Z** is the matrix of covariables, including age and sex (see Additional file 1: Table S1); $\text{a}$ is the corresponding effect; and **e** is the vector of residual effects.

We fitted this model for each gene in each tissue using the least squares approach with R [44] and then ranked all of the genes based on their t-statistics (i.e., ${\upbeta }/\text{SE}$). We defined the top 10% of genes as tissue-specific. We conducted the functional enrichment analysis of tissue-specific genes using the R package clusterProfiler [42].

### PheWAS of candidate genes in humans

To explore whether the orthologues of candidate genes detected for wool and growth traits in sheep were associated with complex traits in humans, we conducted a PheWAS for each of these genes based on the human GWAS data in the GWASATLAS database [45, 46]. Briefly, we explored the GWAS summary statistics of 1299 complex phenotypes from 277 human GWAS (sample size > 5000). We considered genes with corrected *P*-values (FDR) less than 0.05 as significant. For visualization, we classified these complex traits into 12 trait domains based on the available knowledge on tissue biology [47].

### GWAS signal enrichment analysis

We applied the sum-based marker-set test approach below using the R package QGG [21, 48] to determine whether the GWAS signals were enriched in tissue-specific genes. We extended 20-kb windows around gene regions to include regulatory variants.

$${\text{T}}_{{{\text{sum}}}} = \mathop \sum \limits_{{{\text{i}} = 1}}^{{{\text{m}}_{{\text{g}}} }} {\text{b}}^{2} ,$$

where ${\text{m}}_{\text{g}}$ is the number of genomic markers within a list of tissue-specific genes and $\text{b}$ is the marker effect from the GWAS. We controlled the marker-set sizes and LD patterns among markers by applying a genotype cyclical permutation strategy as described previously [49, 50]. To obtain an empirical *P*-value for a list of tissue-specific genes, we repeated the permutation procedure 10,000 times and applied a one-tailed test of the proportion of random summary statistics greater than that observed [21, 48]. We corrected the *P*-values for multiple testing by the FDR method.

## Results

### Estimation of genetic parameters for wool and live weight traits

The number of animals included in the estimation of genetic parameters ranged from 5208 to 7135 depending on the trait analyzed, among which 4713 animals had phenotypic records for all six traits and 1217 animals had genotypes. To use the information from the animals with phenotypes but without genotypes, we used a single-step BLUP (ssBLUP) to estimate the genetic parameters for the six traits. The estimates of the heritability for these traits ranged from 0.05 to 0.36, with MFD and LW having the highest estimated heritability, i.e. 0.36 (SE = 0.04) and 0.33 (SE = 0.03), respectively, and CVFD and CN having the lowest heritability, i.e. 0.05 (SE = 0.02) and 0.07 (SE = 0.03), respectively. The details of the phenotypic records and estimated genetic parameters for the six traits are in Table 1.

In general, the phenotypic correlations among the wool traits and LW were weaker than their genetic correlations. The strongest positive phenotypic and genetic correlations were observed between GFW and LW, with correlation coefficients of 0.50 and 0.77, respectively. MSL was genetically positively correlated with MFD, LW, and GFW, with moderate correlation coefficients of 0.29, 0.29, and 0.27, respectively. It should be noted that LW was negatively genetically (*r* = − 0.44) correlated with CN. The details of phenotypic and genetic correlations among those traits are summarized in Table 2.

**Table 2:** Genetic correlations (above the diagonal) and phenotypic correlations (below the diagonal) between wool traits and live weight in Merino sheep

Trait	MFD (µm)	CVFD (%)	CN (/2.5 cm)	MSL (cm)	GFW (kg)	LW (kg)
MFD (µm)		− 0.21 (0.20)	0.11 (0.18)	0.29* (0.09)	0.06 (0.10)	0.14 (0.09)
CVFD (%)	− 0.07* (0.01)		0.06 (0.31)	0.02 (0.21)	0.05 (0.20)	0.05 (0.19)
CN (/2.5 cm)	0.05* (0.01)	− 0.13* (0.01)		− 0.18 (0.17)	− 0.25 (0.17)	− 0.44* (0.15)
MSL (cm)	0.15* (0.01)	− 0.04* (0.01)	− 0.12* (0.01)		0.24* (0.09)	0.29* (0.08)
GFW (kg)	0.05* (0.01)	− 0.09* (0.01)	0.02 (0.01)	0.18* (0.01)		0.77* (0.05)
LW (kg)	0.08* (0.01)	− 0.09* (0.01)	0.02 (0.01)	0.11* (0.01)	0.50* (0.01)	

Standard error (SE) is presented in parentheses, and * indicates correlation coefficients that were significant (*P* < 0.0.5). MFD, mean fibre diameter; CVFD, coefficient of variation of the fibre diameter; CN, crimp number; MSL, mean staple length; GFW, greasy fleece weight; LW, live weight

### Weighted single-step genome-wide association study

**Fig. 1:**
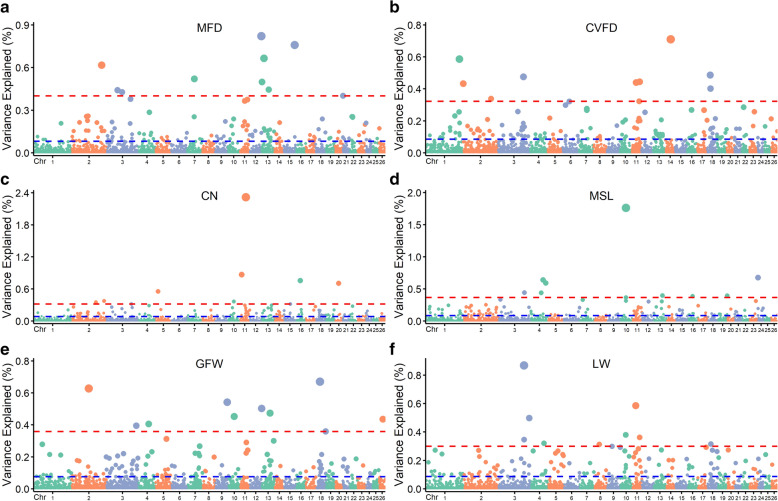
GWAS results of five wool traits and live weight in Merino sheep. **a**–**f** show the mean fibre diameter (MFD), coefficient of variation of the fibre diameter (CVFD), crimp number (CN), mean staple length (MSL), greasy fleece weight (GFW), and live weight (LW), respectively. Each dot represents one window region of 20 consecutive SNPs. The X-axis represents 26 autosomes, and the Y-axis represents the percentages of the genetic variance explained by the windows

As determined via the sliding window strategy with WssGWAS [17], which is a commonly used approach in animal genetics [16, 51], the top 10 genomic regions that explained the largest genetic variance are reported as the QTL for each trait. The percentages of genetic variance explained by windows of 20 consecutive SNPs along the genome for the six traits are shown in Fig. 1. The total genetic variances explained by the top 10 ranked windows ranged from 3.52% (MSL) to 6.94% (CN) across the six traits. In total, we detected 54 unique QTL for the six traits, which cover 81 annotated genes on 21 ovine autosomes, among which six QTL were shared by at least two traits, which indicates that the corresponding causal variants likely exert pleiotropic effects on multiple traits. For instance, the region on *Ovis aries* chromosome 13 (OAR13) between 55,897,362 and 56,044,564 bp was associated with MSL and GFW, and the region on OAR16 between 31,883,408 and 31,963,084 bp was associated with CN and MSL. Detailed information on these 54 QTL is summarized in Additional file 2: Table S2.

To explore the independence of these QTL, we estimated the linkage disequilibrium (LD, *r*^2^) patterns between SNPs along their physical distances in the studied population (see Additional file 3: Figure S1) and found that *r*^2^ decreased to 0.4 when the averaged distance between SNPs increased to 100 kb. Thus, we estimated the LD between adjacent QTL regions separated by less than 100 kb (see Additional file 2: Table S2). A first example concerns two adjacent regions on OAR10, 421,64,611–42,373,451 bp and 42,375,920–42,553,316 bp, which had an averaged LD (*r*^2^) of 0.81, and four haplotype blocks were observed in the combined regions (see Additional file 4: Figure S2a). The second example concerns two adjacent regions on OAR18, 22,741,289–22,871,683 bp and 22,871,683–23,005,179 bp, which had an averaged LD (*r*^2^) of 0.64, and six haplotype blocks were observed in the combined regions (see Additional file 4: Figure S2b). By comparing these 54 regions with the Sheep QTLdb [39, 52], we found that five QTL had been previously reported, whereas the remaining 49 QTL were newly discovered in this study (see Additional file 5: Figure S3a) with most of them (n = 30) being associated with wool traits.

We conducted gene ontology (GO) enrichment analysis for genes within the top 1% of the windows for each trait according to the explained genomic variance (see Additional file 5: Figure S3b and Additional file 6: Table S3). Out of 28 unique enriched GO terms, 15 were related to cell development, and five to metabolic processes. For instance, genes associated with CN, including *RARG*, *NKX2-6*, *PTK2B*, *RARA*, *SOX8*, *STAT3* and *MRE11*, were enriched in the negative regulation of apoptotic processes (*P* = 0.018). Genes associated with GFW, including *MAFG* and *MAFF*, were enriched in the regulation of epidermal cell differentiation (*P* = 0.035).

### Identification of trait-related tissues and cell types by GWAS signal enrichment

**Fig. 2:**
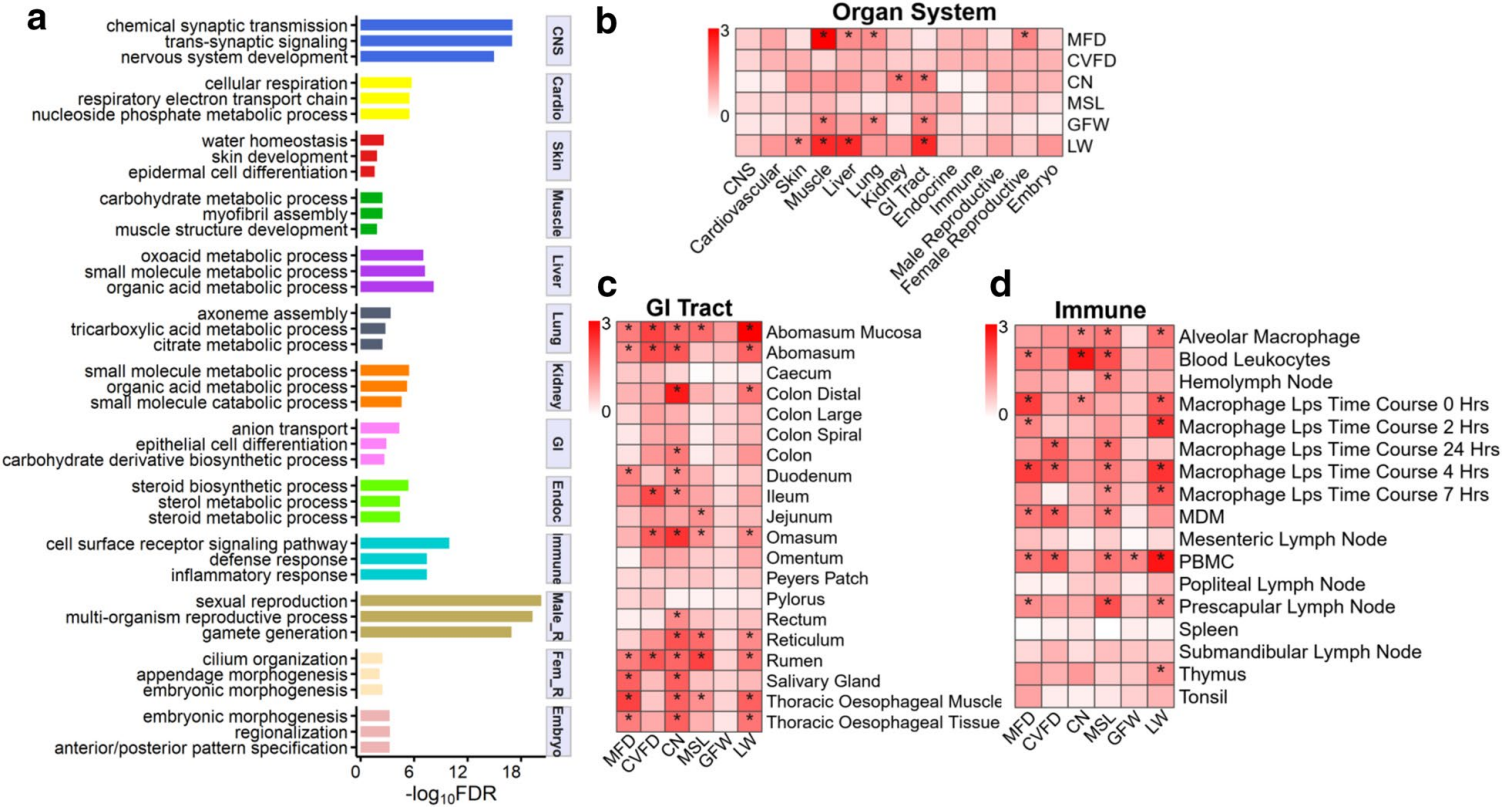
Detection of tissues and cell types related to wool traits and live weight in sheep. **a** Functional enrichment analysis of tissue-specific genes (the top 10% of genes based on *t*-statistics) for 13 organ systems (central nervous system (CNS), cardiovascular system (Cardio), skin, muscle, liver, lung, kidney, gastrointestinal (GI) tract, endocrine (Endoc), immune system, male reproductive system (Male_R), female reproductive system (Fem_R), and embryonic system). **b**–**d** Relationships between the six economic traits and the 13 organ systems, GI tract tissues and immune tissues, respectively. The colour corresponds to the enrichment degree (i.e., − log_10_*FDR*), which was computed by sum-based GWAS signal enrichment analysis based on the top 10% tissue-specific genes and a 20-kb extension. *corrected-*P* (FDR) < 0.05

We found that tissues and cell types within the same organ system were highly positively correlated based on their expression profiles (see Additional file 7: Figure S4), which indicated a high similarity in their tissue-specific expression. The function of these tissue-specific genes clearly agreed with the known biology of the corresponding tissues (Fig. 2a) and (see Additional file 8: Table S4). For instance, brain-specific genes were significantly enriched for the chemical synaptic transmission (FDR = 8.55E−18); skin-specific genes were significantly enriched for water homeostasis (FDR = 0.002) and skin development (FDR = 0.01); immune-specific genes were significantly enriched for immune responses (FDR = 8.56E−11); and liver-specific genes were significantly enriched for organic acid metabolic processes (FDR = 6.35E−9) (Fig. 2a).

To detect the relevant organ systems for each trait, we conducted a GWAS signal enrichment analysis of genes that were specifically expressed in each of the 13 organ systems (Fig. 2b) and (see Additional file 9: Table S5). We found that muscle was significantly (FDR < 0.05) associated with MFD, GFW and LW, whereas liver was significantly associated with MFD and LW. It is worth mentioning that the GI tract was significantly associated with CN, GFW and LW. To further determine which tissues within the GI tract were related to these traits, we conducted a GWAS signal enrichment analysis of genes specifically expressed in each tissue of the GI tract (Fig. 2c) and (see Additional file 9: Table S5). We found that the abomasum mucosa and rumen were significantly associated with five of the six traits (Fig. 2c) and (see Additional file 9: Table S5). Although the entire immune system was not significantly associated with any of these traits, several immune cell types were significantly associated with most of the traits (Fig. 2d). For instance, peripheral blood mononuclear cells (PBMC) and macrophages were significantly associated with LW, and blood leukocytes were the top immune cells associated with CN (Fig. 2d). In addition, macrophages at the early stage (2–7 h post-infection) but not at the late stage (24 h) after LPS infection were significantly associated with LW and MFD (Fig. 2d).

### Expression profiles of candidate genes across multiple tissues

**Fig. 3:**
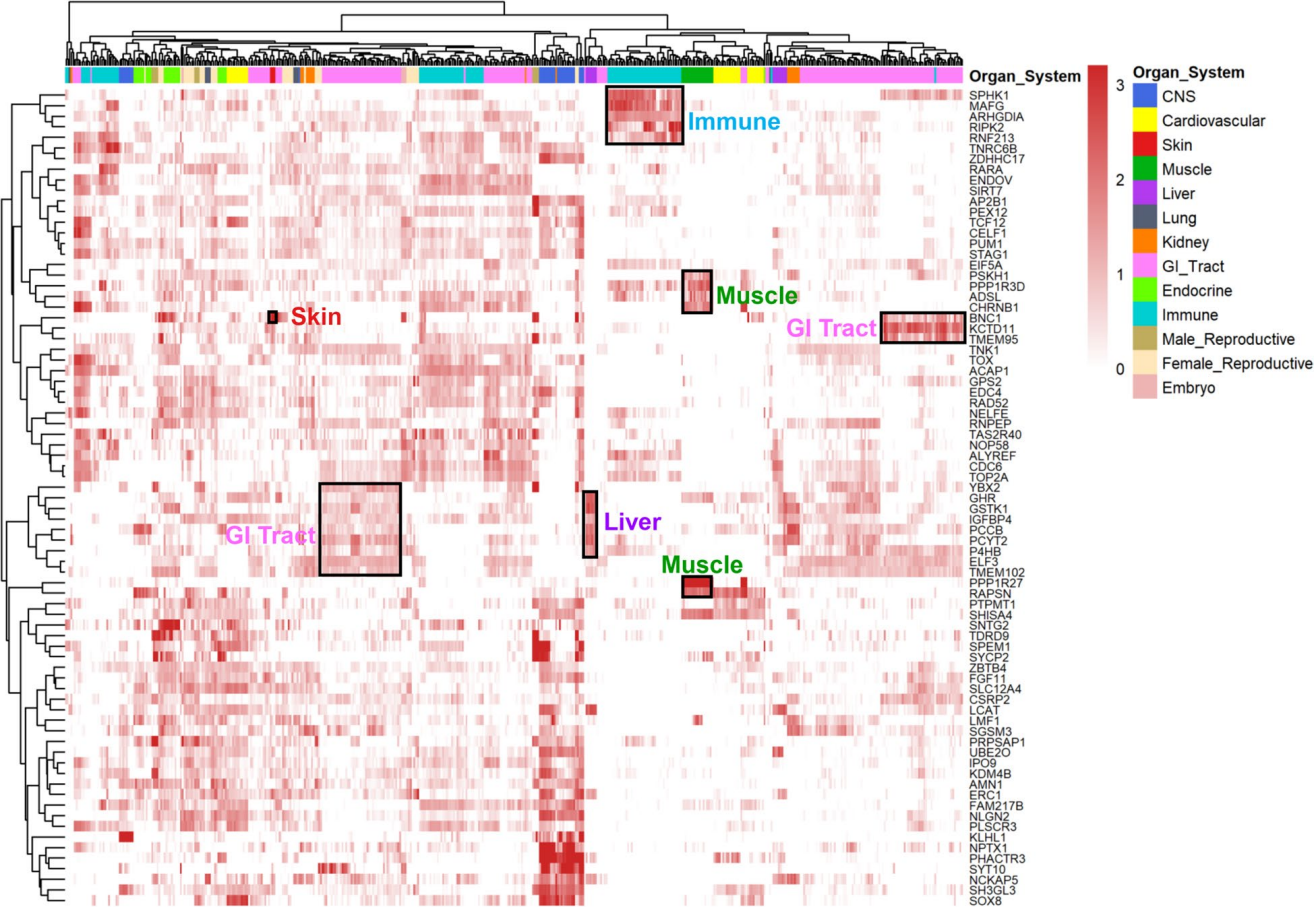
Heatmap of 77 of 81 candidate genes based on the sheep expression atlas. The gene expression levels are normalized as transcripts per million (TPM). The colour corresponds to the log_10_ (TPM + 0.25) value. The Y-axis represents the 77 candidate genes, and the X-axis represents the 13 organ systems

We explored the gene expression patterns of 81 candidate genes across all 87 tissues and cell types (Fig. 3) and (see Additional file 10: Table S6) [43]. The genes *BNC1*, *ENDOV* and *RARA* were specifically and highly expressed in the skin and were candidate genes for MFD, CVFD and CN, respectively. Seven genes, *PPP1R3D*, *ADSL*, *CHRNB1*, *PPP1R27*, *RAPSN*, *SHISA4* and *PSKH1*, were specifically and highly expressed in muscle and were candidate genes for MSL, LW, GFW and MFD. In addition, the expression of several genes exhibited strong immune specificity, such as the *SPHK1*, *MAFG*, *ARHGDIA* and *RIPK2* genes, which were candidate genes for LW and GFW. Three genes, *BNC1*, *KCTD11* and *TMEM9*, were specifically and highly expressed in the GI tract and were candidate genes for MFD and LW. *GHR, IGFBP4* and *GSTK1* were specifically and highly expressed in the GI tract and liver and were candidate genes for CN and MSL.

### PheWAS of candidate genes in humans

**Fig. 4:**
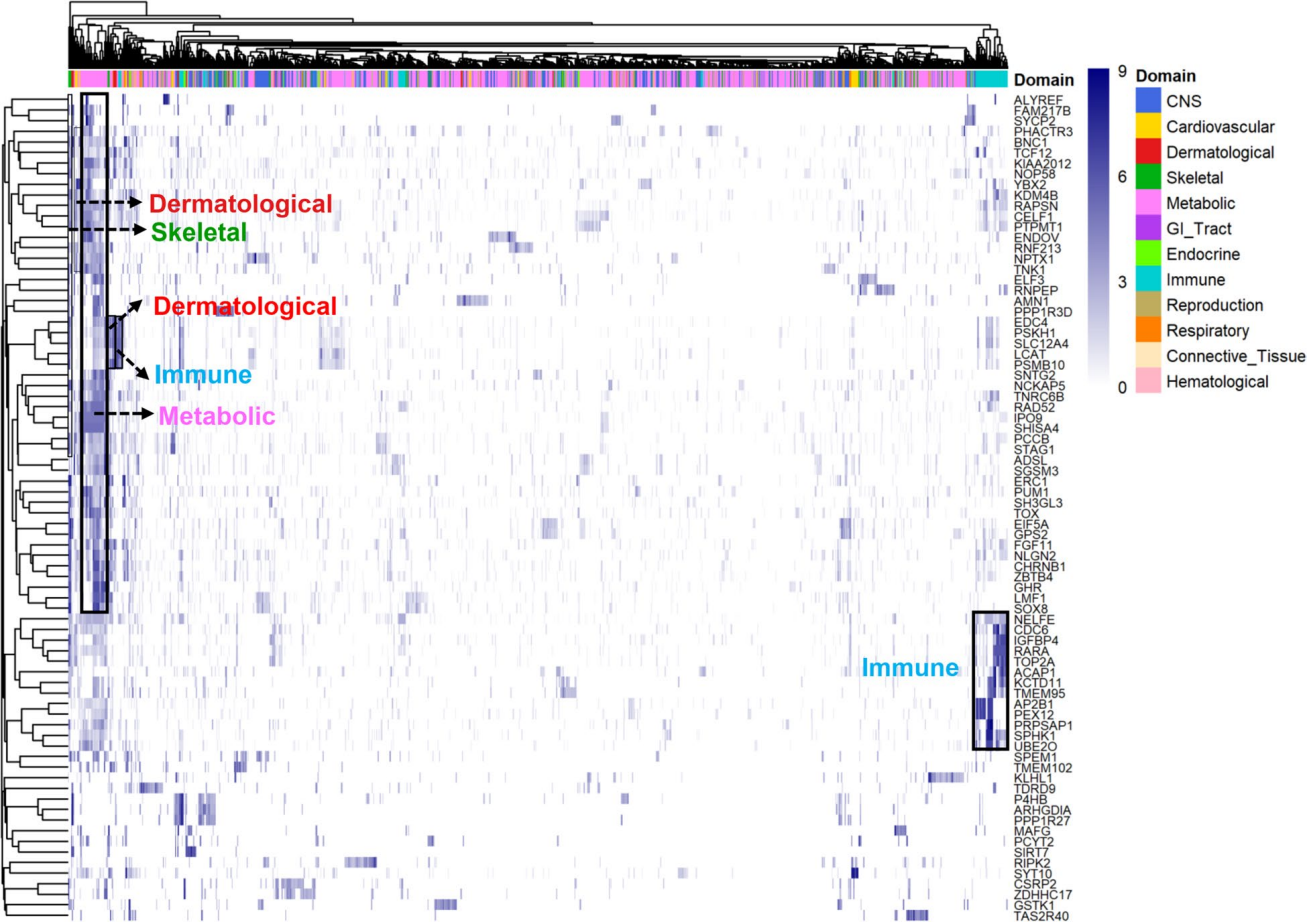
Heatmap of 78 of 81 candidate genes based on the results of the phenome-wide association study (PheWAS). The colour corresponds to the − log_10_*P*-value value from the PheWAS results. The Y-axis represents the 78 candidate genes, and the X-axis represents the 12 trait domains

**Fig. 5:**
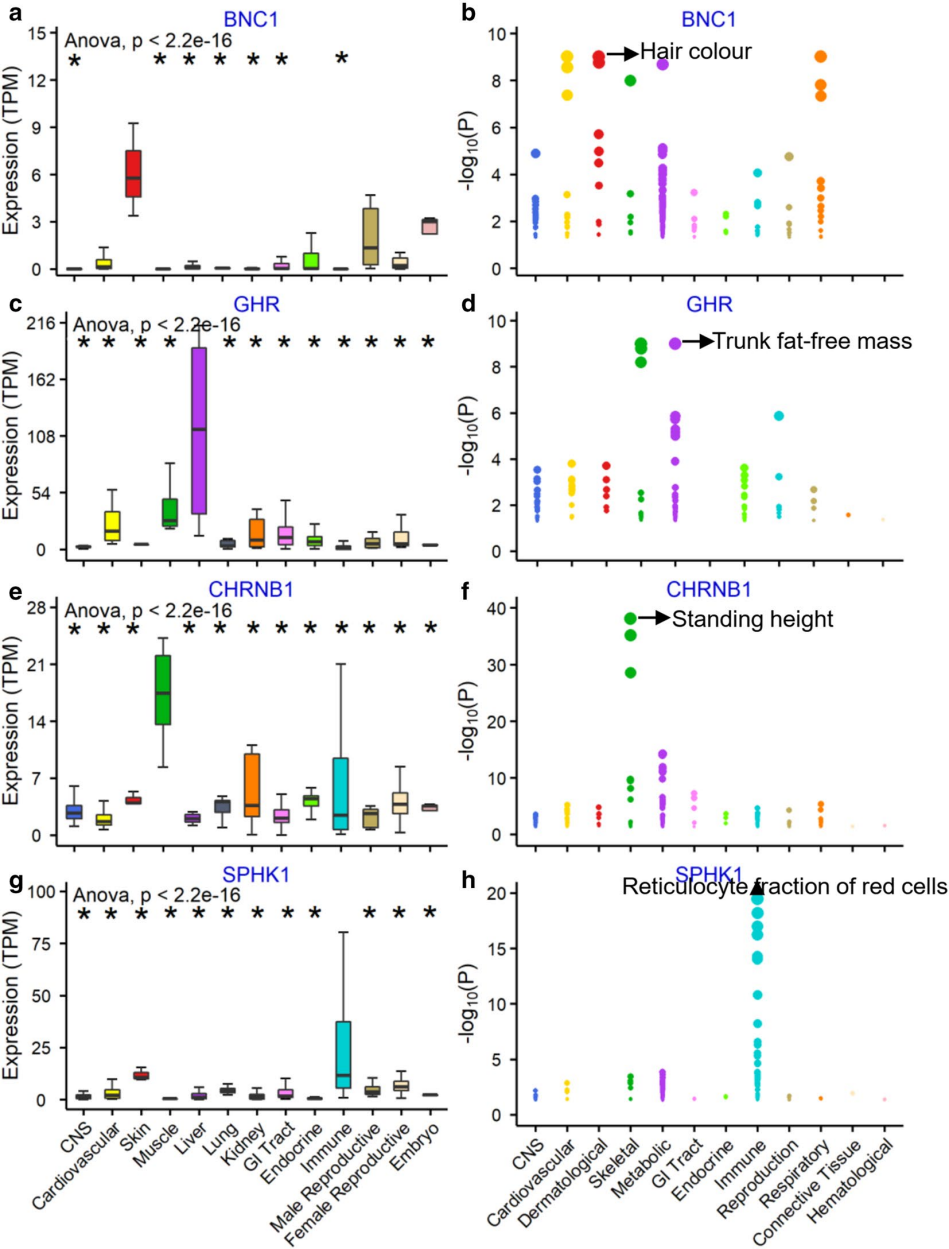
Expression patterns and results of the phenome-wide association study (PheWAS) for four candidate genes. **a**, **b** *BNC1*; **c**, **d** *GHR*; **e**, **f** *CHRNB1*; **g**, **h** *SPHK1*. In **a**, **c**, **e** and **g**, the Y-axis represents gene expression (TPM), and the X-axis represents the samples in 13 organ systems. In **b**, **d**, **f** and **h**, each dot is one trait. The Y-axis represents the -log_10_*P*-value value from the PheWAS results, and the X-axis represents 12 trait domains

The basic assumption is that, among mammals, orthologous genes have similar functions. To explore the potential functions of these 81 candidate genes in humans, we used the human GWAS atlas to conduct PheWASs [45, 46], and this atlas included 1689 traits of 12 trait domains from 322 different GWAS studies (total sample size > 5000) (see Additional file 11: Table S7). As shown in Fig. 4, multiple candidate genes were significantly (FDR < 0.05) associated with similar traits in humans. For instance, genes associated with wool traits in sheep were also significantly associated with dermatological traits in humans, including *ENDOV* (associated with CVFD and hair colour in sheep and humans, respectively), *EDC4* (CVFD and baldness) and *PSKH1* (CVFD and baldness). Several genes associated with wool traits were also significantly associated with immune-related traits in humans, such as the reticulocyte fractions of red blood cells (*SPHK1*) and white blood cells (*NPTX1*) (Fig. 4), which is consistent with previous findings that indicated that the immune system is involved in hair follicle development [53, 54]. Many genes associated with LW in sheep exhibited significant associations with traits related to metabolism and skeletal tissues in humans, such as *ADSL* (body mass index), *RAPSN* (height), *PSKH1* (height), *PPP1R3D* (standing height), *SHISA4* (heel bone mineral density), *ADSL* (standing height) and *CHRNB1* (body mass index). Four candidate genes, *BNC1* (associated with MFD in sheep), *GHR* (CN and MSL), *CHRNB1* (LW) and *SPHK1* (LW), are shown as examples in Fig. 5 and are specifically expressed in the skin, liver, muscle and immune system. Considering these results together with those from the sheep expression atlas, we propose 10 candidate genes for wool and LW traits in sheep (Table 3).

**Table 3:** Candidate genes for wool traits and live weight in Merino sheep

Trait	Gene	Chr	Start	End	Window rank	Specifically expressed tissue (*P*-value^a^)	Specifically associated domain (*P*-value^b^)
MFD	*BNC1*	18	22,758,417	22,784,473	TOP3	Skin (1.6E− 09)	Dermatology (2.0E− 15)
CVFD	*ENDOV*	11	51,243,368	51,257,814	TOP10	Skin (0.03)	CNS (1.0E− 5)
CN	*CDC6*	11	40,114,668	40,125,179	TOP1	Embryonic (< 2.2E− 16)	Immune (2.1E− 09)
CN	*RARA*	11	40,151,341	40,172,203	TOP1	Skin (0.04)	Immune (2.5E− 14)
CN	*IGFBP4*	11	40,250,021	40,260,382	TOP1	Liver (6E− 13)	Immune (8.4E− 12)
CN & MSL	*GHR*	16	31,832,933	32,000,445	TOP3 & TOP9	Liver (< 2.2E− 16)	Metabolic (3.1E− 17)
MSL & GFW	*PPP1R3D*	13	55,922,753	55,923,652	TOP7 & TOP5	Muscle (< 2.2E− 16)	Immune (0.002)
LW	*CHRNB1*	11	26,753,567	26,761,779	TOP2	Muscle (< 2.2E− 16)	Skeletal (8.8E− 39)
LW	*ADSL*	3	215,669,356	215,684,701	TOP3	Muscle (< 3.5E− 14)	Metabolic (9.2E− 14)
LW	*SPHK1*	11	54,357,681	54,359,873	TOP4	Immune (< 2.2E− 16)	Immune (3.4E− 20)

Chr, chromosome; MFD, mean fibre diameter; CVFD, coefficient of variation of the fibre diameter; CN, crimp number; MSL, mean staple length; GFW, greasy fleece weight; LW, live weight; CNS, central nervous system; GI tract, gastrointestinal tract

^a^The *P*-values were computed by comparing the expression (TPM) of the given gene in the target tissues with that in the remaining tissues

^b^The results were obtained from a phenome-wide association study (PheWAS), and the *P*-values were obtained from the top significant traits within the target domains

## Discussion

Genetic improvement of wool traits and LW is an essential goal in the sheep breeding industry. In this study, we did not directly measure carcass composition traits but recorded LW at the age of 15 months, which indirectly reflects the health and meat production of the sheep [55]. Compared with the results reported by Di et al. [56], we found slightly higher estimates of heritability for MFD (0.36 vs. 0.22), GFW (0.28 vs. 0.17) and LW (0.33 vs. 0.23) and slightly lower estimates for MSL (0.27 vs. 0.32) and CVFD (0.05 vs. 0.09) based on an average sample size of 2639. The heritabilities of these traits were lower than those estimated in Australian Merino [57, 58]. We found a highly favourable genetic correlation between LW and GFW (*r* = 0.77) and moderate positive genetic correlations between MSL and LW (0.28), MSL and GFW (0.27) and MSL and MFD (0.29), which is consistent with previous studies [55, 59]. In addition, the global LD pattern between SNPs in this population was consistent with that previously reported by [60].

We detected multiple novel QTL for wool traits that were not in the sheep QTLdb [39]. Most of the QTL included in the sheep QTLdb originate from a single previous study that was conducted using a classical single-marker GWAS with 50 K SNPs in a relatively small population (n = 765) [9]. Here, we performed a WssGWAS to jointly analyse all available data (n = 7135), including data from individuals with both phenotypes and genotypes and data from individuals with phenotypes but without genotypes. Gutierrez-Gil et al. [3] reported five genomic regions under positive selection in Australian Merino, among which two were detected as QTL in our study, i.e. the QTL on chromosome 11 for LW and the QTL on chromosome 15 for MFD. Gutierrez-Gil et al. [3] also reported 52 genes within these five positive selection regions, among which 12 were detected as candidates for wool traits or LW in our study, including *AMN1* for CVFD and MSL, and *CHRNB1* and *YBX2* for LW. These results provide evidence that the region that is associated with the positive selection signal in Merino might be related to wool phenotypes. The remaining different results between these studies could be due to differences in the statistical methods used, and/or to differences in allele frequencies and in patterns of QTL segregations between the populations analysed [3, 11].

Comparison of the data from the sheep expression atlas and human PheWAS data revealed that several candidate genes showed tissue specificity and conserved functions among mammals. Multiple candidate genes were associated with immune traits in humans, which is in line with previous findings showing that the immune system has been involved in the development of hair follicles [53, 54, 61]. For instance, it has been demonstrated that the immune system mounts a specific autoimmune response against hair follicle antigens [53], and that the breakdown of the hair follicle immune privilege leads to T-cell inflammation [54]. These results reveal the importance of integrating omics data for the detection of relevant tissues and candidate genes for complex traits [21, 62–64] and suggest the potential of cross-species mapping to benefit the livestock industry and human biomedicine [65, 66]. However, it should be noted that the PheWAS results based on human data, similar to the results from comparative genomic mapping, are not easily transposable to findings in livestock, and that their interpretation is often biased by many factors that differ between humans and livestock. Therefore, the results from a PheWAS suggest biological hypotheses that require further validation.

*BNC1*, a candidate gene for MFD, encodes a zinc finger protein that is present in the basal cell layer of the epidermis and in hair follicles and plays a regulatory role in keratinocyte proliferation [67]. It is mainly expressed in the outer root sheath in hair follicles [68]. The gene *IGFBP4*, which was located in the top QTL of CN, explained 2.32% of the genetic variance. Based on the gene annotation in GeneCards [69], *IGFBP4* is involved in the regulation of cell growth, glucose metabolic processes, and insulin-like growth factor receptor signalling. Previous studies have demonstrated that *IGFBP4* is preferentially expressed in hair follicles [70, 71] and that it functionally interacts with *IGF1*, which plays a key role in the development and growth of hair follicles [72, 73]. Therefore, we consider that *IGFBP4* is a strong candidate gene for CN. *CHRNB1* is involved in muscle contraction and muscle fibre development [74] and is specifically and highly expressed in muscle. The human PheWAS results showed that *CHRNB1* was significantly associated with skeletal traits. A previous GWAS conducted by Kominakis et al. [75] also revealed that *CHRNB1* is associated with body size in sheep. Thus, we consider that *CHRNB1* is a promising candidate gene for LW. It should be noted that 22 QTL regions did not contain annotated genes and it would be interesting to link these regions to target genes using a more precise functional annotation of the sheep genome, such as that developed by the Functional Annotation of Animal Genomes (FAANG) project [76, 77].

## Conclusions

We estimated the genetic parameters for five wool traits and live weight in a dual-purpose Merino sheep and detected 81 candidate genes for these six traits using a WssGWAS approach. By integrating multiple biological datasets (e.g., the sheep expression atlas and PheWAS) with GWAS signals, we propose a list of the 10 most promising candidate genes for these traits: *BNC1*, *ENDOV*, *CDC6*, *RARA*, *IGFBP4*, *GHR*, *PPP1R3D*, *CHRNB1*, *ADSL* and *SPHK1*. Our findings shed light on the genetic and biological basis of wool traits and live weight, and provide valuable information for genome selection in Merino sheep.

## Supplementary Information

**Additional file 1: Table S1.** Details of the summary statistics for individual samples of ovine transcriptomes. The transcriptome data were obtained from Clark et al. [43].

**Additional file 2: Table S2.** Summary of the top 10 regions and their corresponding candidate genes for wool traits and live weight in Chinese Merino sheep. Chr, chromosome; start (bp); end (bp); var (%), percentage of the genetic variance explained by the window region; mean linkage disequilibrium (LD) estimate (r^2^); genes, genes located within the window region. –, no genes were located in the window region or in the top 10 ranked windows for the corresponding trait. The six traits in this study were: mean fibre diameter (MFD), coefficient of variation of the fibre diameter (CVFD), crimp number (CN), mean staple length (MSL), greasy fleece weight (GFW) and live weight (LW).

**Additional file 3: Figure S1.** Dynamics of the linkage disequilibrium (LD, r^2^) of SNPs along their distances. The R-square (r^2^) was lower than 0.4 (moderate LD) when the average distance between SNPs was approximately 100 kb. The R-square (r^2^) decreased rapidly as the marker distance increased.

**Additional file 4: Figure S2.** Haplotype blocks and pairwise linkage disequilibrium (LD) of adjacent QTL regions. (a) SNPs located within 421,64,611–42,373,451 bp and 42,375,920–42,553,316 bp on OAR10; (b) SNPs located within 22,741,289–22,871,683 bp and 22,871,683–23,005,179 bp on OAR18. The black triangle blocks are haplotype blocks. The values in the boxes are pairwise SNP correlations (D’), whereas the boxes without numbers indicate complete LD (D’ = 1). The colour in the box corresponds to the pairwise SNP correlation.

**Additional file 5: Figure S3.** General characteristics of the candidate genes across the six traits analyzed. (a) Comparison of our newly discovered QTL with previously known QTL in the sheep QTLdb; (b) Enriched gene ontology (GO) terms (biological processes, BP) for genes within the top 1% of the windows in each trait.

**Additional file 6: Table S3.** Top five enriched gene ontology (GO) terms (biological processes, BP) for genes within the top 1% of the windows for each trait. The six traits analysed in this study were: mean fibre diameter (MFD), coefficient of variation of the fibre diameter (CVFD), crimp number (CN), mean staple length (MSL), greasy fleece weight (GFW) and live weight (LW).

**Additional file 7: Figure S4.** Correlation clustering of 87 tissues and cell types within 13 organ systems based on the expression of their genes. The top colour corresponds to the correlation coefficients, and the bottom colour corresponds to the organ systems.

**Additional file 8: Table S4.** Enriched gene ontology (GO) terms (biological processes, BP) for the top 10% of genes expressed in specific tissues based on the *t*-statistic ranks in each organ system.

**Additional file 9: Table S5.** Significant GWAS signal enrichment of complex traits across all tissues within organ systems, the gastrointestinal (GI) tract and the immune system.

**Additional file 10: Table S6.** Expression of 77 of 81 candidate genes based on the sheep expression atlas. The gene expression levels were normalized as transcripts per million (TPM).

**Additional file 11: Table S7.** Results of the phenome-wide association study (PheWAS) for 78 of 81 candidate genes.

## Data Availability

The genotypes and phenotypes used in the current study were generated from commercial farms and are not publicly available. The sheep expression atlas was obtained from [43], and the human PheWAS data are available from [45].
