# Supplementary figures and images for: Integration of a single-step genome-wide association study with a multi-tissue transcriptome analysis provides novel insights into the genetic basis of wool and weight traits in sheep
Source: Genet Sel Evol. 2021 Jun 30;53:56. doi: 10.1186/s12711-021-00649-8 (PMC8247193; doi:10.1186/s12711-021-00649-8)

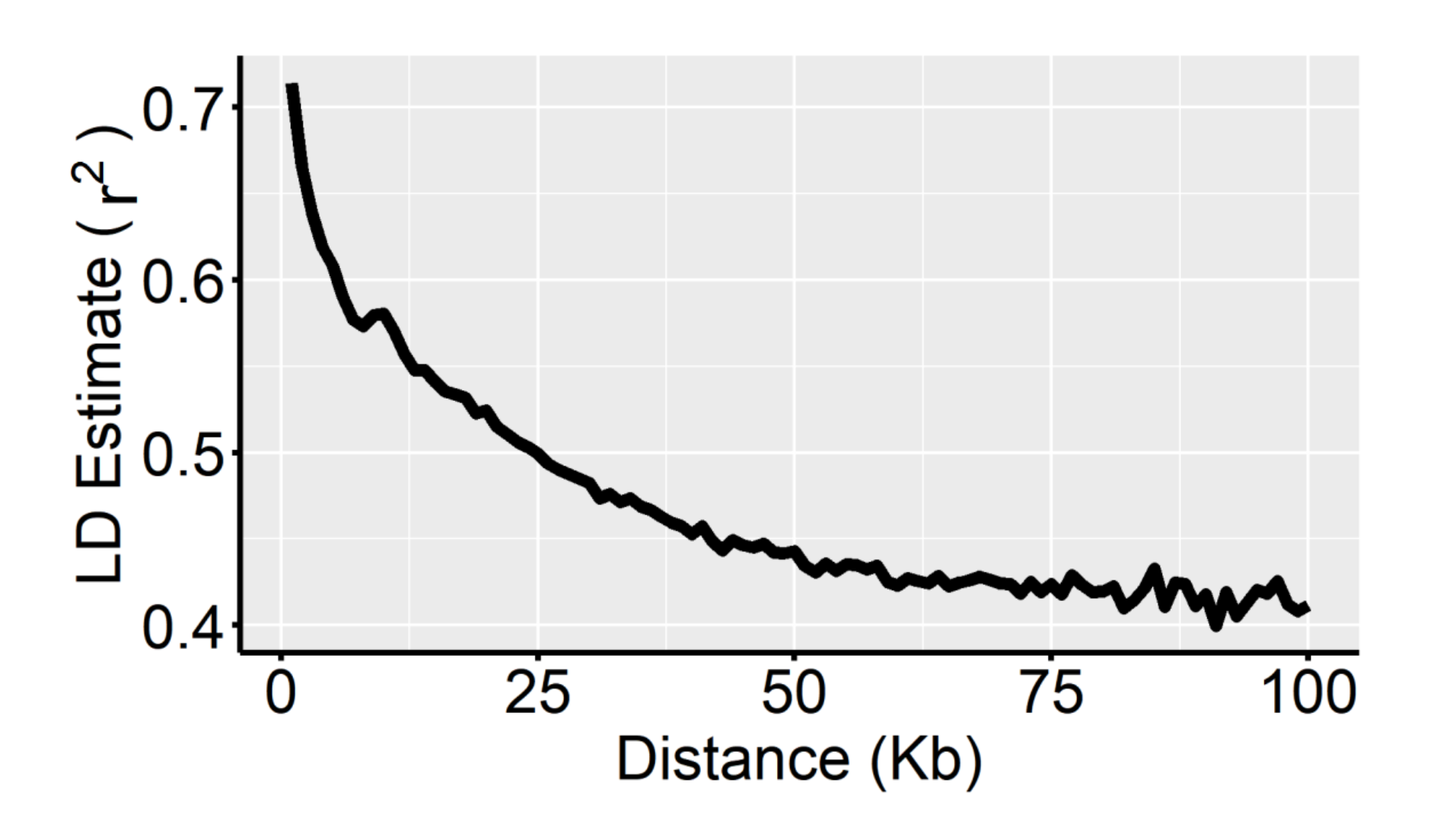

Supplement: Supplementary file 3 — Additional file 3: Figure S1. Dynamics of the linkage disequilibrium (LD, r2) of SNPs along their distances. The R-square (r2) was lower than 0.4 (moderate LD) when the average distance between SNPs was approximately 100 kb. The R-square (r2) decreased rapidly as the marker distance increased. [file 12711_2021_649_MOESM3_ESM.tif]

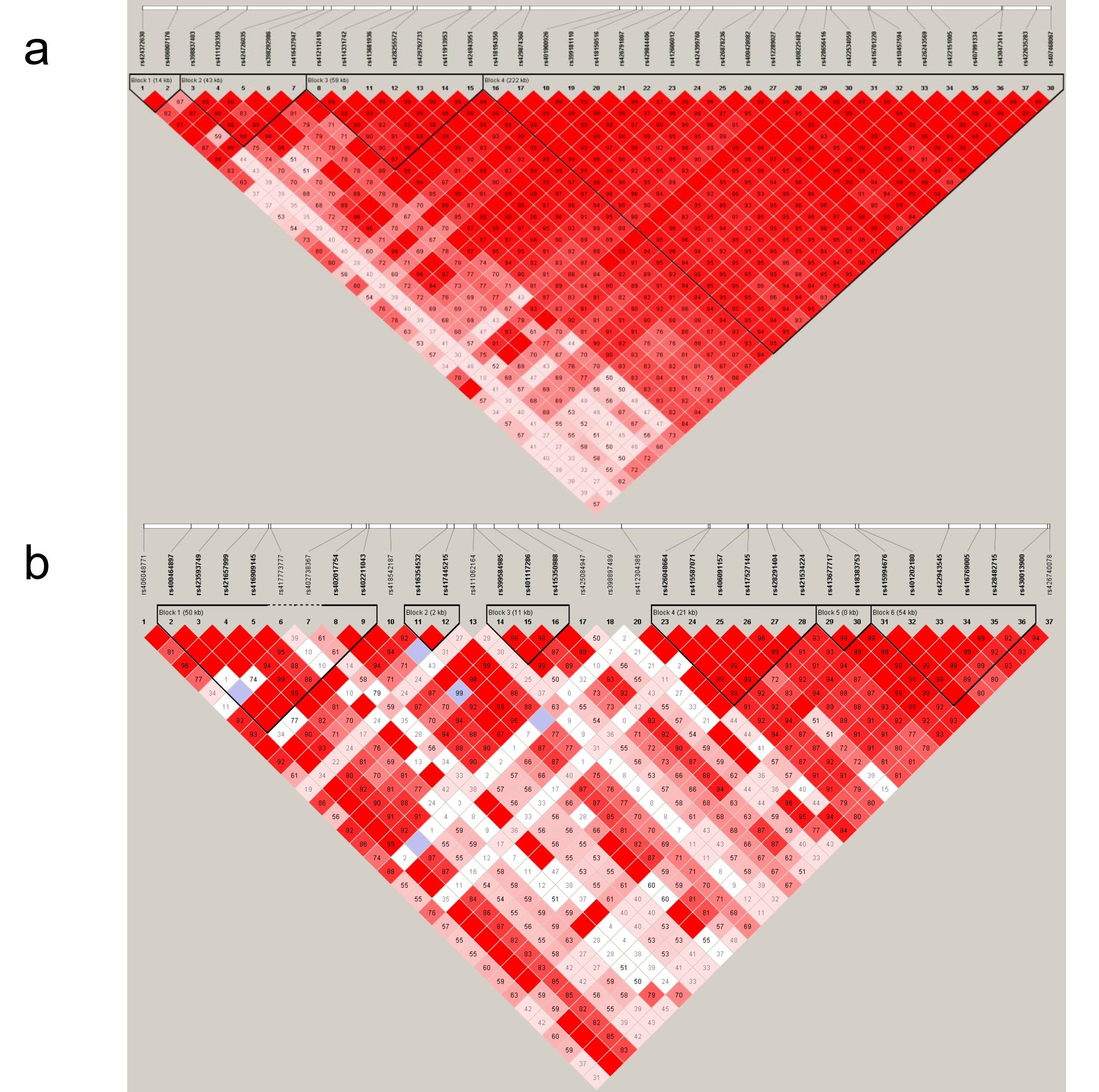

Supplement: Supplementary file 4 — Additional file 4: Figure S2. Haplotype blocks and pairwise linkage disequilibrium (LD) of adjacent QTL regions. (a) SNPs located within 421,64,611–42,373,451 bp and 42,375,920–42,553,316 bp on OAR10; (b) SNPs located within 22,741,289–22,871,683 bp and 22,871,683–23,005,179 bp on OAR18. The black triangle blocks are haplotype blocks. The values in the boxes are pairwise SNP correlations (D’), whereas the boxes without numbers indicate complete LD (D’ = 1). The colour in the box corresponds to the pairwise SNP correlation. [file 12711_2021_649_MOESM4_ESM.tiff]

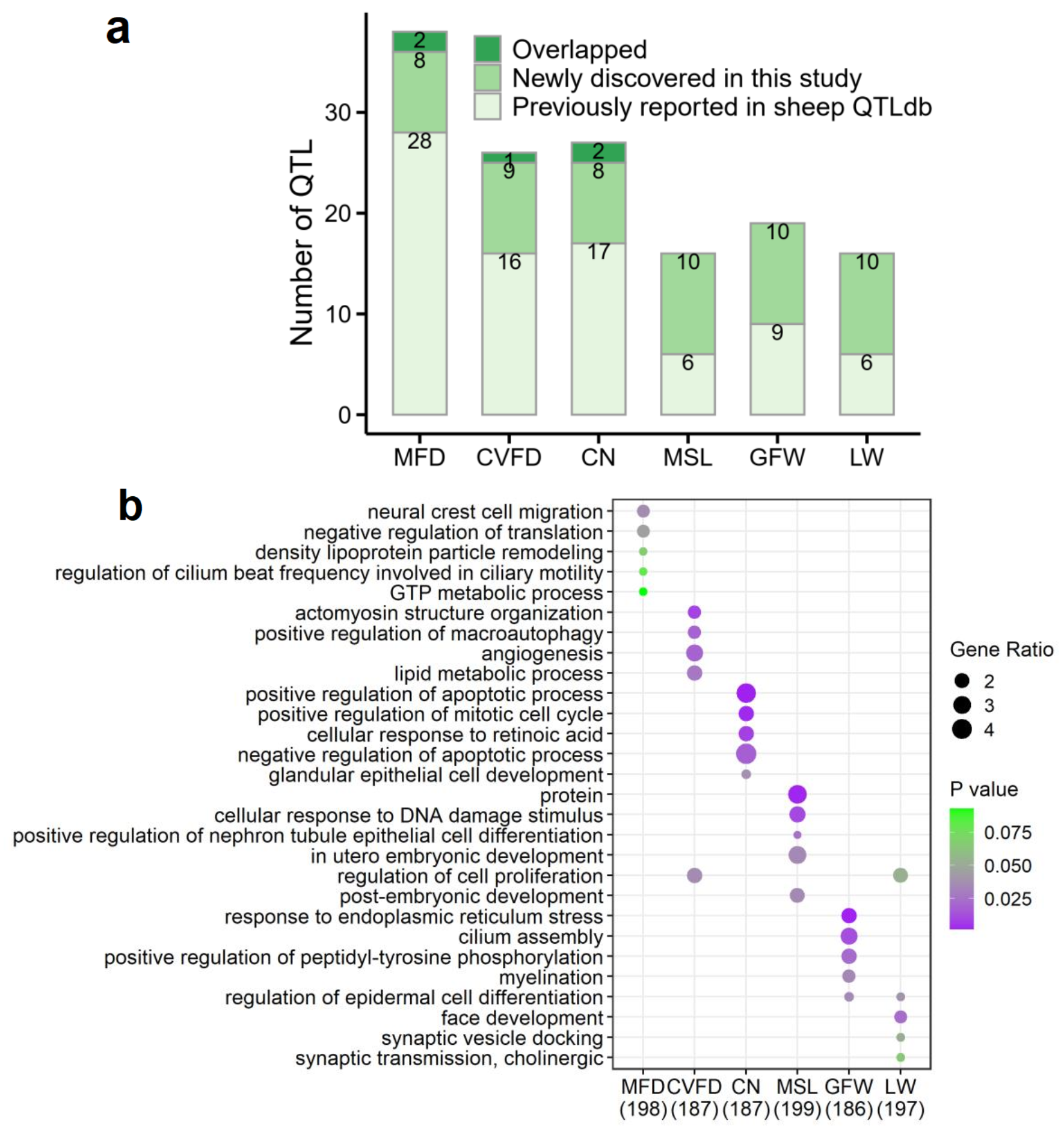

Supplement: Supplementary file 5 — Additional file 5: Figure S3. General characteristics of the candidate genes across the six traits analyzed. (a) Comparison of our newly discovered QTL with previously known QTL in the sheep QTLdb; (b) Enriched gene ontology (GO) terms (biological processes, BP) for genes within the top 1% of the windows in each trait. [file 12711_2021_649_MOESM5_ESM.tiff]

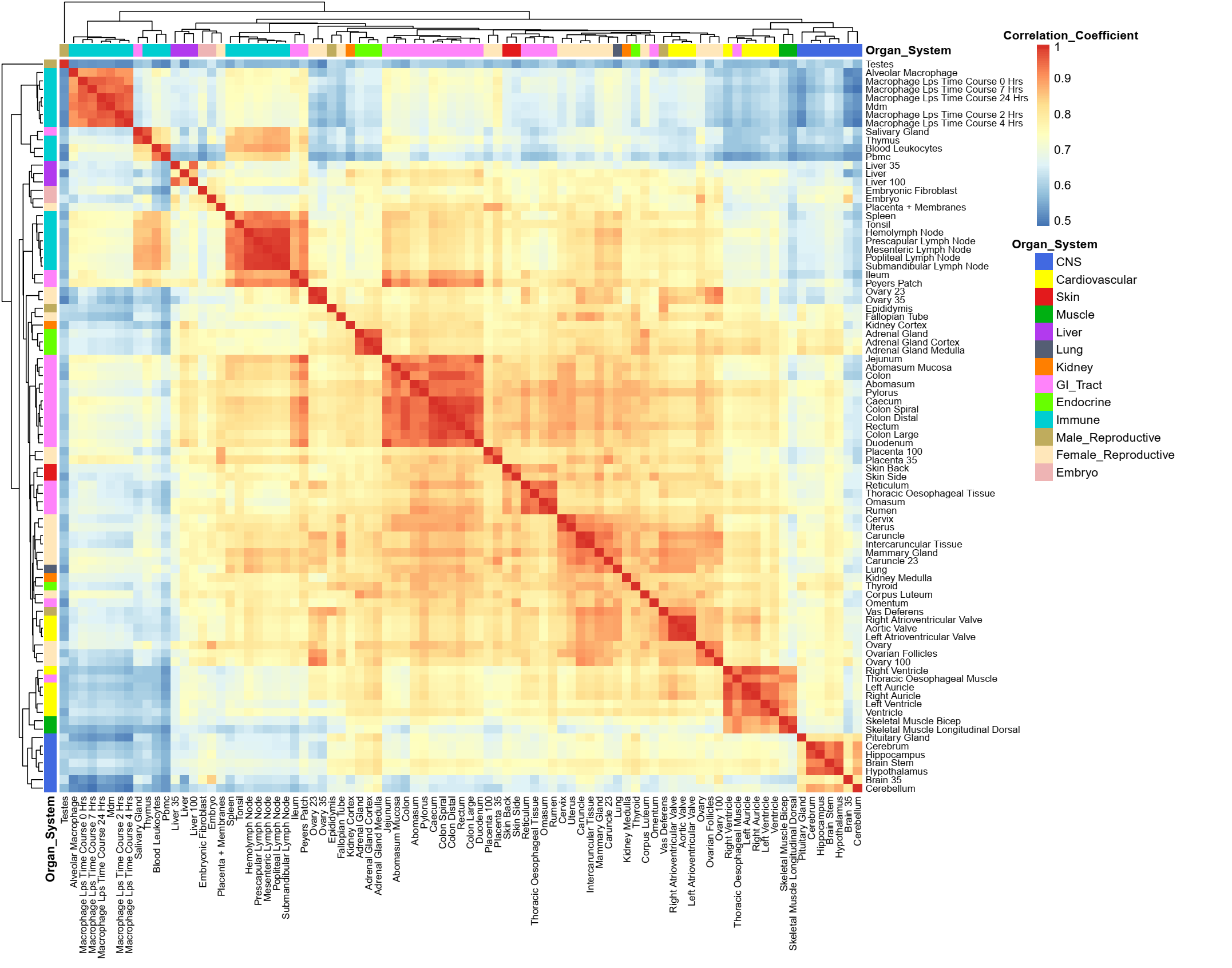

Supplement: Supplementary file 7 — Additional file 7: Figure S4. Correlation clustering of 87 tissues and cell types within 13 organ systems based on the expression of their genes. The top colour corresponds to the correlation coefficients, and the bottom colour corresponds to the organ systems. [file 12711_2021_649_MOESM7_ESM.tiff]
